# American Academy of Dental Science

**Published:** 1895-06

**Authors:** 

**Affiliations:** American Academy Dental Science


### Reports of Society Meetings.

### AMERICAN ACADEMY OF DENTAL SCIENCE.

The regular monthly meeting of the American Academy of Dental Science was held at Young's Hotel, Boston, Wednesday evening, December 5, at six o'clock, President Smith in the chair.

The paper for the evening was read by Dr. H. F. Libby, of Boston. Subject, "A Method of inserting Gold Fillings with the Use of Hand-Burnishers."

(For Dr. Libby's paper, see page 325.).

### DISCUSSION.

*President Smith.*—Gentlemen, it is not only a great pleasure, but a great profit to meet a man of strong individuality,—a man who not only thinks, but puts his thoughts into practical shape. That is what Dr. Libby has done in presenting to us this evening a very practical paper on a very practical subject. I, for one, feel deeply grateful for the manner of its presentation, and I feel that I echo the sentiments of all in making this statement.

Perhaps it would be fitting that Dr. Fillebrown, who has been freely quoted by the essayist, should open the discussion on this subject. Although no arrangements have been made with Dr. Fillebrown to do so, I trust he will make the opening remarks.

*Dr. Fillebrown.*—I have been very much interested in the paper. It is divisible into two parts. One may be called the development of the plugger, and the second its application.

The Harvard pluggers have been considerably quoted to-night, and as I had something to do with their evolution, it is proper for me to speak of the manner in which they were evolved. At the time that Dr. Shumway first introduced the ivory points, I was as much interested in the matter as Dr. Libby. I am glad to have my memory refreshed in regard to the time that has elapsed since then,—I was not aware how long ago it was. The first knowledge that I had of Dr. Shumway's using ivory points was from Mr. Hood, who was then travelling. He came into my office one day, and in the course of conversation made the remark that Dr. Shumway was filling teeth with ivory points by rubbing the gold in, and that a smooth steel point would answer the same purpose.

I was at the time operating for a lady patient, and left her to speak with Mr. Hood. There was a cavity which I had just finished excavating on the distal surface of the left inferior bicuspid, which was extremely hard to get at. I was at that time using the mallet exclusively, it being then in vogue. I had decided before seeing Mr. Hood that that cavity was a place where I could not use the mallet, and had almost come to the conclusion that I must fill it with something besides gold. I said to Mr. Hood, "I have a cavity in which I can try the method of which you speak," and I immediately took an old excavator and broke off the end to about the right size, smoothed it down with a file, sand-papered it, and finished by polishing with pumice-stone, so that I had a tolerably smooth surface. I did not stop to temper it, but went right to work to fill the cavity at hand. I do not believe it was thirty minutes after Mr. Hood first spoke of this method to me before I had that cavity filled. Fifteen years afterwards I examined that filling, and found it to be as perfect as when first put in. Instead of using Dr. Shumway's method of rubbing the gold, I applied the force as Dr. Libby mentioned,—namely, carrying the gold to place and turning the instrument a little, and at the same time giving it a gradual pressure.

I did not burnish in each piece, for the reason that if you rub gold too much with a steel instrument you will injure its surface.

After placing in the above-mentioned filling I at once made a set—a half dozen to begin with—of those smooth points; laid aside my mallet, and for years afterwards used that method. Dr. Shumway sent me a set of twenty-four of his ivory points, and I did some good work with them, but was never fully satisfied: they were too frail and weak. Finally I made patterns for a set of pluggers, which I sent to Philadelphia and had made in steel, tempered just as hard as they could be, and I put in fillings with those, even in the incisors, and they gave me great satisfaction. I remember one case where there was a crescent decay in the cutting-edge of a lateral incisor. I contoured this with gold, and for years it was doing well, and the result was that I led several of my friends to adopt this method. I had five or six sets made of these smooth points in Philadelphia, and they were distributed around among my friends, but they did not have such good luck with them as I, and later had them serrated. Unless one was very careful in using these smooth points, especially in contouring a surface where there was nothing to hold the gold, the pluggers were very likely to slip, and it was here that my friends found their greatest difficulty.

After a time the effort to prevent the slipping began to tire me, and I followed in the footsteps of others and had some very fine serrations put on the oval surfaces, and have used them ever since with great success. That is the evolution of the Harvard plugger.

One point that I wish to call attention to is the fact that the Harvard plugger was never intended for use with a mallet, and no filling which has been exhibited here shows the work of the plugger in any proper way. In using those oval points with a mallet you will not, of course, get the gold evenly packed, so I cannot admit it to be a fair test of the work of the plugger to show a filling which has been put in with a mallet. The proper way to use those points is with hand-pressure, and when you bring down the pressure roll the plugger a little, and that force will condense the whole surface of the piece of gold you have just put in. I don't know how a filling made in that way would look under the microscope, but you cannot expect the best work from the Harvard plugger when used with a mallet.

I want to say that I am pleased with this set of pluggers that has just been passed around. They answer a want that I have repeatedly felt, and I thank Dr. Libby for the form of handle which he has given us, as it is just what I need.

The statement which Dr. Libby quoted from me, that you must not rub gold at all, was made in early days. I know perfectly well that it can be done, and the Herbst method is an excellent way to fill teeth. It is particularly useful in starting fillings. When you wish to be positively certain that the gold is in close contact with the surface of the cavity, take some soft gold and, as I put it, just let it "smell the flame;" lay in sufficient gold to loosely fill the cavity, and upon this a piece of cotton, and rub the cotton into the cavity with a burnisher; you will be surprised to see what a wonderful adaptation there is of gold to the surface; then add more in the same way until you have a good foundation upon which you can pack your gold by pressure. This soft gold should not be absolutely non-cohesive, but such a kind as can be readily made a little cohesive,—enough to give it a kid-like feeling and softness. It is a non-cohesive gold, and yet has cohesiveness enough to make it maintain its connection with the rest of the filling; in this you have one ideal filling. Dr. Libby's is another ideal filling which, from the illustrations he has shown, seems to possess great merit, and I certainly thank him for bringing before us to-night his method in a manner which has been so plain; and I certainly want to know more about it by observation of the operation itself.

*Dr. Andrews.*—I want to say how much I have enjoyed Dr. Libby's paper. I know how earnestly he has been at work, for I see the evidence of it, and it seems to me that other members ought to take up some special subject and work it up in this way if necessary. I believe in the power of pictures to show what one means. I have worked at that thing myself and have done a great deal of micro-photography. I would like to ask Mr. Smith what power was used in making these photographs.

*Mr. Smith.*—The objects were magnified in the original photographs seventy-five to one hundred times, and as they were cast on the screen at a distance of forty feet, the pictures represented an enlargement of about five hundred times.

*Dr. Andrews.*—Thank you, I have enjoyed the views very much. I have used the old ivory points, and I believe I have done some good work with them, though I have not used them much of late years. I confess that I could not always make the pieces of gold stick,—I mean in working this way. I tried this method at Dr. Libby's office and it worked well, and I have ordered a part set of these instruments. If Dr. Clapp were here he would commend them, I am sure, as he has had some little experience with them. I think that if it were only for presenting this style of wooden handles, Dr. Libby has done a good thing for the profession. He has given us a set of instruments with which we can do our work easier and better than with the old steel handles.

*Dr. Williams.*—Dr. Libby has excellently demonstrated one principle, which is a very old one and one which was often referred to by Dr. Joshua Tucker, and that was the difference between the gradual, steady pressure and a jerk. He illustrated it in this way: we will suppose a man to be a good operator, but he is nervous; he puts his gold in and presses it into place by a sharp jerk. Another man takes a little and puts on gradual pressure, and the filling made in the latter way will undoubtedly be more durable. Dr. Joshua Tucker was one who established a reputation for a solid gold filling, and it was partly by that method, on that principle, that he attained his excellence. He also made use of the principle that was alluded to by Dr. Fillebrown, that of slightly annealing the gold. I learned from one of his pupils that it was considered a secret by him in those times.

I am very glad that Dr. Libby has so admirably illustrated this principle, which, in the hands of Dr. Tucker, helped to give the first success and reputation to gold fillings.

*Dr. Wiksell.*—I have two reasons for wishing to speak: one to thank the Academy for the chance of hearing this paper, and again, to speak a little in endorsement of the method. I think we all by this time have recognized that there is a Libby method; that no matter what its evolution has been, we have had some distinct and original ideas presented to us. I have had considerable success with this method where I have had faith to use it. Not having Dr. Libby's instruments, I have used the common burnishers and the corkscrew pluggers with universal success. So far I have been waiting to see a filling come back broken off. A bicuspid which I have filled has been standing the force of mastication for some two months. I felt when it went out of the office that the patient might be back within a week, but I saw the filling to-day, and it was as solid as ever.

One point that the doctor did not call attention to is this, that he uses a bur slightly larger than the size of the plugger which he proposes to use in putting in the filling.

*Dr. Gerrish.*—As one of the guests of your society, I want to thank you for the privilege of being here. For thirty years I have followed one line of practice, and in that particular method I have had very satisfactory results. I am a user of Abbey's non-cohesive No. 4 foil. I adopted it at first, and have used it constantly from that time to the present. My method has not been what Dr. Libby describes as a burnishing method, though I have inserted all my fillings with hand-pressure, and the force has always been exerted with the sides or angles of the instruments. I have continued in that line of work for the reason that I have been, modestly speaking, satisfied with the results.

I became acquainted with Dr. Libby at his home in Wolfboro', New Hampshire, and we have spent many pleasant hours together in fishing, hunting, and talks on dentistry. He described his method, and told me that if I would come down to his office he would show me the whole system. I was very much interested, the more so because a short time before I had repaired a filling for the wife of an Episcopal clergyman, a patient of his, which excited my curiosity, as it contained no pits. This fact led me to consult him, and he kindly showed me his method of operating, which I adopted at once.

I don't believe there is any other form of gold or method of work that will save a tooth as perfectly as non-cohesive foil. I am old-fashioned in that. I respect the work of the leading operators of other methods and have had a chance to see their fillings. I think I have worked after as many eminent operators as perhaps any man in this room, and I have studied the fillings, trying to find out, if possible, the method that the dentist used. My definition of a perfect filling is one which makes the best adaptation to the cavity or the most perfect adjustment to the walls of the tooth. It should also be hard enough to withstand the force of mastication, and it should be easily put in. My method, I claim, has the first two advantages. So far as the latter is concerned, it is not easy to do it,—it requires a good deal of work, and it differs from Dr. Libby's method in that I use the sides of the instruments, while he uses the point of the instrument to fasten the gold in place, and then the instrument is turned and becomes a burnisher, so that each new piece extends a little bit beyond the original piece. Now, the adaptation of that extension is perfect, because the piece is very small. I admit that many of the operators who use the mallet get beautiful, solid fillings, but the trouble is with some they don't fit the cavity. I have seen fillings that you could take out and find to be a perfectly solid mass of gold, but they were not worth a picayune for saving teeth. When a piece of gold is once moved from the place that you put it, it isn't worth anything for the saving of the teeth. In Dr. Libby's method the extension of the gold is so minute and it is so carefully pressed into place that there is a perfect adaptation to the walls of the cavity, and the success of the filling is assured.

For the last thirty years I have used the non-cohesive foil, using for the past two years Dr. Libby's method for finishing and contouring.

*Dr. Dodge.*—For quite a number of years my time has been almost exclusively occupied at the operating-chair; although I have achieved a fair amount of success, I have been uncomfortably aware of the deficiencies of my operations. Until recently my method of inserting gold fillings has been largely with the automatic plugger and cohesive gold, and many times have I noticed a lack of adaptation to the walls of the cavity; and many times a disintegration of tooth-substance at the margin of the cavity. In the course of my inquiries I found Dr. Libby was apparently getting the results I desire to attain, and I determined to learn more of his method. I received an invitation from him to attend a clinic at his office last June. To say that I was pleased with the operation that was there performed only expresses it mildly. My eyes were opened to possibilities in the working of gold of which I had not dreamed; I felt that what Dr. Libby had done I could probably do, and I ordered from him at that time a complete set of his instruments. On my return to my office I felt it was almost impossible for me to go back to the old system of filling; I therefore went to work and made burnishers from such instruments as I could readily procure, and when I say that from that time until the present I have inserted only burnished gold fillings (where gold has been used), you can form some idea of my feelings in regard to this form of filling. And, furthermore, I wish to say that I have no hesitancy in my friends examining these fillings with a powerful magnifying-glass. Speaking of the magnifying-glass, I am strongly wedded to it in examination of the teeth for cavities and in the excavation and preparation of the same. Cavities which formerly I considered thoroughly excavated when examined with a mouth-mirror of small magnifying power, such as are furnished by the dental depots, when subjected to the use of this magnifying-glass very often show imperfections which can be remedied.

I wish to say that I believe I have found in the method of Dr. Libby a system of filling that by the exercise of due care and a strong determination to overcome all obstacles will save the teeth of our patients better than any method that I have ever before seen used.

I have not found much difficulty in picking it up with these instruments highly burnished. When I received the set of instruments which I requested Dr. Libby to have made for me, the first thing I did was to examine them thoroughly with this powerful magnifying-glass. I noticed in them as they came from the manufacturers a good many scratches and indications of unevenness. I polished them off with a chamois-skin buff, using crocus; then I made a buff of cotton flannel, and, using rouge for a finish, I got as high a polish as I could possibly get upon them. That is the condition they are in when I am using them, and I believe that I can do a great deal better work by having them highly polished than as they were when sent by the manufacturers. There is not so much possibility of little particles of steel working into the gold. I never use them a second time without giving them buffing at least with the cotton flannel, and sometimes with the chamois.

*Dr. Wiksell.*—I have been trying to convince Dr. Libby of the use of an assistant. A sharp-eyed girl, I calculate, saves just one-third of the time for an operator. My assistant picks up the gold with a platinum point, anneals it, and puts it right where I want it. I timed Dr. Libby one day while he was operating, and in one column I put down the number of seconds that it took him to pick up his gold, anneal it, and put it where he wanted it, and in another column I put down the number of seconds that he actually occupied in burnishing, and on adding up the two columns of figures it was found that he had spent thirty-three and one-third per cent. of his time in picking up and annealing the gold. Therefore I estimate that he could do with an assistant two hours' more work a day. I had this drummed into me by a neighbor, a brother dentist, and I am going to drum it into other men,—that to work with an assistant is the greatest saving. You don't have to take your mind off the operation after the girl is trained in what you want her to do, and she earns her pay twice over every day.

*Dr. Werner.*—I have the pleasure of being an invited guest this evening, and it is a privilege that I much appreciate. I am very much in favor of what Dr. Gerrish has said. Still, he comes short of my ideas.

*Dr. Gerrish.*—I do of my own, doctor.

*Dr. Werner.*—For I want to continue to full contour. I think he has the right ideas in the use of non-cohesive gold, but I wish to use the matrix in order to thoroughly condense it and finish with cohesive gold, bringing the filling out to full contour; then I think we have the ideal conditions. I would ask Dr. Libby if he has ever used tin-foil in his method?

*Dr. Libby.*—No, sir.

*Dr. Werner.*—I find tin-foil an excellent article for use at the cervical wall, and I believe that our best results come from using tin-foil and gold at that part, say in proportion of one-half of tin to one of gold. I am a thorough believer in gold; I believe when we work it right with tin at the cervical wall it will do more to save teeth than any other filling-material, and I use it in every possible case. Of course in many cases the cements are the best preparatory filling-material. As to amalgam I have a very poor opinion of it and a very limited use for it. It is a pity we never had illustrations of how gold can be condensed at the cervical wall when surrounded by a proper and firmly held matrix. At that point our fine operators have failed. They did good work and made excellent solid cohesive gold fillings; their failures were at the cervical wall, and how could it be otherwise? When you put retaining-points and pits at the cervical wall where the enamel and dentine concentrate in thin layers, and then hammer cohesive gold against that, how could you expect the tooth would stand it? How different when a matrix is placed there, and your non-cohesive gold is thoroughly condensed: then we get a perfect adaptation and strengthen the weak surfaces.

The greater part of a cavity, say four-fifths, I fill with non-cohesive gold, finishing with cohesive rolled gold of No. 40 or 60. General principles we should hold fast to. It does not matter how you condense, with serrated or non-serrated or with wooden points. I believe the essayist has the vital principle in his method and I will be glad to see him operate very soon.

*Dr. Fillebrown.*—There seems to be a misunderstanding in regard to the surface of the gold that was shown in these pictures. I understood that the filling was put into this cavity and then removed, and the picture shows the surface that was adjusted to the bottom of the cavity.

*Dr. Libby.*—Did not I mention that the ivory slab was divided into halves? That was a serious omission, as it is quite important to know that in order to understand what surface was exposed to the microscopic lenses. It was divided in halves and bolted, and after each filling was put in and finished the bolts were removed, and when we parted it the filling dropped out.

In relation to using tin at the cervical wall, I saw one case that came from an advocate of this method in New York, and it disappointed me so much that I never undertook to use it in my practice. I cannot see why it is not just as well to pack soft gold at the cervical wall, if you have instruments with which it can be done properly, and in my estimation it is much better, for you certainly avoid the discoloration, and not only that, but there are some conditions of the mouth that will dissolve tin, and you are not taking any chances of that kind when you use gold.

*Dr. Andrews.*—Some have expressed a desire to know what kind of gold Dr. Libby uses.

*Dr. Libby.*—I used for fifteen years, exclusively, Hood & Reynolds No. 3 foil, which is an extra soft cohesive gold, and it always gave me great satisfaction. I knew there was going to be a time when I should show this method before the societies, and I went to work two years ago experimenting with the various foils, and I found no difficulty in using any kind of foil as far as the working of the gold was concerned. To day I am using Williams's electric foil. It comes beautifully folded for my use. I am now using No. 40,—I began with No. 30, but I did not find substance enough in that and so changed to No. 40, which seems to suit me very well. I have tried rolled gold, and that can be worked just as well. I do not see any benefit from using the rolled gold, but there is no difficulty in working any of them by this method, and I would say that No. 3 foil, the electric, and the rolled, all produce a fine finish.

*Dr. Andrews.*—You say in regard to these bars of gold they were filled into a form of ivory which was then taken apart and the filling taken out.

*Dr. Libby.*—Yes, the same surface was shown in every picture; they were all taken from the sides of the different fillings.

*Dr. Andrews.*—How would it do to fill in, by these different methods, against a surface like glass,—an absolutely perfect surface?

*Dr. Libby.*—That is not practical in our every-day work. However, as the same surface is shown in every case, it is just as fair for one method as for the other. In the mallet-bar you saw a great many patches, plainly exhibiting what phenomenon occurs in trying to mallet a piece of gold. I have been asked a great many times how will these fillings wear? Several people have said, "I don't think they will stand attrition or the amount of work that will come upon them." Well, I have had no difficulty whatever in that respect, and it is a curious fact that away back at the time of my failures, not only with soft foil but with cohesive foil, the failures were up where I could not reach them,—at the coronal surface they seldom failed, and apparently it was because I did not have the proper instruments to reach the bottom of the cavities that I failed in my early work.

*Dr. Wilson.*—I noticed that some of the larger instruments that

Dr. Libby has passed around are very slightly serrated,—are they not?

*Dr. Libby.*—Yes, they are serrated for one purpose only,—that of picking up the gold. Let me tell you that the large instruments I do not use for burnishing; they are simply made for packing soft gold.

*Dr. Cook.*—I would ask Dr.Libby if the cavity into which these bars of gold were filled was smaller at the top than it was at the bottom?

*Dr. Libby.*—Yes, it was something of a pear shape.

*Dr. Cook.*—In such a case I should not expect that you would get as good a result from the mallet as you would from burnishing in with your points which were fitted and shaped for that kind of a cavity, and I do not consider that it is hardly a square test for the mallet. I should not think of packing cohesive gold into that kind of a cavity, I should prefer to have it larger at the top than at the bottom.

*Dr. Libby.*—That is a form of cavity that I should not consider universally practical,—square walls. I believe in spherical surfaces, and if I used straight walls, it would be a departure from the way in which I usually prepare my cavities. My usual practice is to have the base of the cavity as rounding as possible.

*Dr. Cook.*—The point I wish to make is that, while a cavity prepared in that manner may be best for filling by your method, it is not adapted for the reception of a filling which is packed in by a mallet. Suppose we illustrate by an ordinary cork stopper,—that is larger at the top than it is at the bottom, and the farther in you drive that cork the more effectually it stops up the mouth of that bottle. Now, if we had a cavity prepared in that manner, as we packed our gold in the pressure applied would carry the gold towards the walls of the cavity, but in trying to mallet a filling into a cavity that was larger at the bottom than at the top, you drive the gold away from the walls of the cavity, and the illustrations on the screen are not a fair comparison, because the cavities are not adapted to the same methods.

*President Smith.*—In regard to Dr. Libby's instruments, which were shown on the screen and also were passed about here, I would say that you can use them as we commonly use the hand-plugger direct, or you may follow the burnishing method described by Dr. Libby. I have used them as hand-pluggers with great satisfaction for some time. The instruments, in themselves, I think are simply grand.

Are there any more remarks? Perhaps Dr. Libby may have something to say in conclusion before we close the discussion of the paper.

*Dr. Libby.*—Just a few words only. I wish to thank the gentlemen for the kind attention which they gave to this paper and for the interest which has been displayed in my method. I do not claim that it has reached perfection, and would suggest that each one who takes interest enough in it to put it in practice should try and discover some new feature that would be of interest to all of us. Dr. Dodge has already started the ball a-moving, and presents an idea that I have not looked into particularly,—that of a more highly polished surface than burnishers are usually finished with as they come from the manufacturers,—and so other gentlemen will discover points, and our combined observations may produce a method of filling which will give entire satisfaction to ourselves and to our patients.

*Dr. Allen.*—May I ask if these instruments are in the market?

*Dr. Libby.*—They are made by the Boston Dental Manufacturing Company, who now have orders for quite a number of sets. I have had great difficulty in getting what I wanted, and there has been some delay in getting them out, but I did not want any of them to go out until they were just right.

*Dr. Eames.*—Thanks seem to be in order, and I wish to express my appreciation of Dr. Libby's paper, and especially the illustrations. We have had many papers in the past whose value would have been enhanced, perhaps, more than one-half by proper illustration. I would move you that the thanks of the Society be extended to Dr. Libby for his valuable paper and illustrations of the method of filling he has presented to us this evening.

Unanimously voted.

William H. Potter, D.M.D.,
*Editor American Academy Dental Science.*

